# Indirect quantification of IgG using a digital refractometer, and factors associated with colostrum quality in Norwegian Red Cattle

**DOI:** 10.1186/s13028-019-0494-9

**Published:** 2019-12-05

**Authors:** Julie Føske Johnsen, Johanne Sørby, Cecilie Marie Mejdell, Åse Margrethe Sogstad, Ane Nødtvedt, Ingrid Hunter Holmøy

**Affiliations:** 1 Section of Terrestrial Animal Health and Welfare, Norwegian Veterinary Institute, Pb 750 Sentrum, 0106 Oslo, Norway; 2 Biri Dyreklinikk, Birivegen 75, PB 44, 2832 Biri, Norway; 3 ANIMALIA, Norwegian Meat and Poultry Research Centre, Lørenveien 38, PB 396, Økern, 0513 Oslo, Norway; 4 Department of Production Animal Clinical Sciences, Norwegian University of Life Sciences, PB 8146 Dep, 0033 Oslo, Norway

**Keywords:** Agreement, Brix%, Calf health, Dairy, Diagnostic test evaluation, Digital refractometer immunoglobulins, Welfare

## Abstract

**Background:**

There is an increased interest in using digital refractometers to indirectly assess colostrum quality of dairy cattle, but knowledge on diagnostic accuracy for Norwegian Red dairy cows is lacking. Recent research has indicated a profound variability in the colostrum quality among dairy cows and herds in Norway. The aim of this study was to evaluate the diagnostic test sensitivity and specificity of a digital refractometer (Brix refractometer) at different cut-offs in Brix% for detection of colostrum of high quality (> 50 g/L) defined by the gold standard single radial immunodiffusion (IgG g/L). Furthermore, we aimed to identify possible associations between selected herd and cow-level management factors and colostrum IgG-levels in Norwegian Red dairy cows.

**Results:**

Median colostrum IgG level across 167 cows from 19 herds was 35.0 g/L, ranging from 5 to 129 g/L. Mean Brix% (± SD) was 19.7 ± 4.12%, ranging from 10.1 to 30.5. Most samples (72.5%) had inferior quality as compared to the international standard of 50 g/L. Brix% and IgG in colostrum were strongly correlated (r = 0.71, P < 0.001). A Brix cut-off of 22%, which is currently recommended, yielded a sensitivity of (95% CI) 69.4% (54.6–81.7) and a specificity of 83.1% (75.0–89.3) for identifying colostrum with high quality (> 50 g/L). The only factor found to be associated with low colostrum quality was parity. Specifically, cows in the second parity were found to produce colostrum with low quality compared to cows in parities four and later.

**Conclusions:**

The agreement between colostrum IgG and Brix% is good. However, the diagnostic test evaluation indicates suboptimal performance in identifying high vs. low colostrum quality in this population, possibly related to a high proportion of the samples with < 50 g/L IgG. The only factor found to be associated with low colostrum quality was parity. Specifically, cows in the second parity were found to produce colostrum with lower quality. Future research should investigate colostrum and serum IgG levels which best prevent calf illness under Norwegian conditions.

## Background

Optimization of calf colostrum management is of utmost importance for preventing impaired welfare and poor health [e.g. 1–3]. In addition to nutritional factors, the first secretion of the mammary gland is rich in immunoglobulins (Ig). One of the most abundant Igs, which are absorbed from the gut upon ingestion of colostrum, is IgG1 (hereafter referred to as IgG). McGuirk and Collins [4] estimated that the calf needs 100–200 g of IgG within 6 h after birth. Consequently, the concentration of IgG in the colostrum is one measure of its quality with regard to prevention of failure of passive transfer of immunoglobulins defined as FPT (< 10 g/L IgG in serum of calves aged 24–48 h). A widely used cut-off level for IgG, which identifies colostrum of high quality is > 50 g/L IgG [5]. Other important determinants of colostrum quality are e.g. bacterial contamination.

The gold standard for determining the colostral IgG concentration is single radial immunodiffusion (RID) [6, 7]. However, on-farm testing of the colostrum quality is of increasing interest. Digital refractometers are already widely used in Norwegian dairy herds. By use of a digital refractometer, the content of total solids in colostrum can be obtained. The unit of measurement is given as Brix%. The correlation between IgG concentration and Brix% is strong, and IgG can therefore be predicted in bovine colostrum based on Brix% readings [e.g. 8–10]. The diagnostic accuracy of the digital refractometer is generally high, but the predictive ability can vary with different prevalence of samples with inferior quality [11]. Moreover, it is essential to evaluate the refractometer in the population in which it is intended to be used [12]. An evaluation of the use of a digital refractometer to estimate colostrum IgG levels among Norwegian Red dairy cows is lacking. For the producers to take informed decisions about colostrum quality, it is important to know how accurately the indirect measure of colostrum quality (Brix%) can identify colostrum of high quality.

Recent studies have found profound variation in the colostrum quality of Norwegian Red dairy cows [13, 14]. Colostrum IgG varies at cow level [15, 16], but also between herds [13]. This variation might therefore be related to management, i.e. feeding, housing and dry period management.

The aim of this study was to evaluate the diagnostic test sensitivity and specificity of a digital refractometer (Brix refractometer) at different cut-offs in Brix% for detection of colostrum of high quality (> 50 g/L) defined by RID (IgG g/L). Furthermore, we aimed to identify possible associations between selected herd- and cow-level management factors and colostrum IgG concentration in Norwegian Red dairy cows.

## Methods

### The herds

Twenty dairy herds in the mid-east of Norway (Hedmark county, Ringsaker municipality), were contacted by telephone at the beginning of the study period which ran from March to September 2016. Inclusion criteria were at least four calvings during the sampling period (May–August 2016), an even distribution of parities among the expected calvings, breed (Norwegian Red) and willingness to participate. One farm was excluded due to an insufficient number of calvings (< four) during the sampling period. One of the authors (JS) conducted two visits per farm; at the beginning and at the end of the study period. During the first farm visit, information regarding general features of the herd, feeding- and pasture management was obtained through an interview using a questionnaire (Table 1). Additionally, a herd level assessment of the cleanliness (rated from 1 to 4 by increasing dirtiness) was performed of all dairy cows’ udders and hind legs as described by Whist and Sølverød [17]. Farmers received all equipment necessary for sampling colostrum and written as well as oral instructions on how to perform the sampling.

**Table 1:** Herd level variables with associated IgG values included in the statistical analysis of possible risk factors for low colostrum quality in 167 colostrum samples collected from 19 herds in a specific geographical region in Norway (Ringsaker municipality, Hedmark County)

Variable	Class or mean (SD)	Herds(N)	Samples (n)	IgG, g/L (SD)
Barn type	Tie stall	2	17	50.4 (9.51)
	Freestall	17	150	39.6 (1.74)
Herd size	49.9 (18.23)	19	167	40.7 (1.84)
Herd milk yield	8314 (751)	19	167	40.7 (1.84)
Weeks prior to calving concentrate is fed to heifers and dry cows	1	4	34	36.7 (21.38)
	2	8	67	37.0 (20.61)
	3	7	66	46.6 (26.79)
Concentrate (kg/days) fed to heifers at expected	1.5	2	19	37.7 (20.09)
Calving	2	5	38	39.7 (20.02)
	2.5	2	20	37.2 (21.79)
	3	8	74	43.6 (23.06)
	3.5	1	8	36.9 (17.43)
	4	1	8	39.9 (37.7)
Concentrate (kg/days) fed to multiparous cows at expected calving	1	1	10	37.3 (19.77)
	1.5	1	9	38.1 (21.64)
	2	6	48	40.4 (24.14)
	2.5	2	20	37.2 (21.80)
	3	7	64	43.6 (24.08)
	3.5	1	8	36.9 (17.43)
	4	1	8	39.9 (37.96)
Pre-partum increment of concentrate (kg/days) heifers	0.16 (0.172)	19	167	40.7 (1.84)
Pre-partum increment of concentrate (kg/days) cows	0.17 (0.171)	19	167	40.7 (1.84)
Solid feed type	Silage round bale	13	117	41.2 (25.93)
	Silage round bale + straw	4	33	40.7 (17.20)
	Total mixed ration	2	17	37.5 (19.16)
Other feed	Potato	4	31	40.3 (24.44)
	Brewers grain	1	10	40.5 (24.17)
	Other (beet pulp, yeast)	1	10	41.2 (24.01)
	Missing	13	116	40.5 (24.17)
Amount other feed (kg/days/lactating cows)	43.4 (24.60)	6	51	41.3 (25.02)
	Missing	13	116	41.5 (41.48)
Pasture	All dairy cows	17	148	40.2 (24.54)
	All pregnant heifers	1	10	43.7 (15.88)
	Pregnant heifers and dry cows	1	9	46.9 (16.69)
Pasture turnout	May	3	23	36.1 (18.79)
	June	14	125	40.0 (22.90)
	July	2	19	51.4 (31. 61)
Pasture access	During day and night	7	66	38.3 (18.94)
	During the day or night	12	101	42.3 (26.37)
Additional feed at pasture?	No	5	43	42.7 (26.4)
	Silage round bale	12	107	40.5 (26.42)
	Total mixed ration	2	17	37.5 (19.16)
Length of pasture period (months)	< 3	8	74	45.6 (26.93)
	3–4	5	45	34.7 (17.90)
	> 4	6	48	38.9 (22.06)
Minerals ad libitum?	Yes	4	32	32.9 (17.16)
	No	15	135	42.6 (24.73)
Complementary minerals to all in the herd?	Yes	16	144	41.0 (22.94)
	No	3	23	39.1 (28.73)
Additional minerals to pregnant cows/heifers?	Yes	8	65	43.2 (26.58)
	No	11	102	39.2 (21.71)
Licking minerals in addition to minerals in feed?	Yes	12	104	42.7 (25.22)
	No	7	63	37.5 (20.85)
Days prior to calving cow is moved to calving pen	1	4	32	38.3 (21.82)
	2	4	37	41.8 (25.05)
	3	3	24	42.4 (17.70)
	4	2	19	46.2 (21.93)
	14	1	9	38.1 (21.64)
	Missing	5	46	39.0 (28.09)
Herd hygiene score	1	1	10	28.9 (11.70)
	1.5	5	48	39.9 (20.84)
	2	9	76	42.4 (26.10)
	2.5	3	23	40.9 (27.76)
	3	1	10	43.7 (15.88)

### Colostrum sampling

Farmers were instructed to exclusively sample cows without signs of health disorders. From each cow, two aliquots of composite colostrum samples (i.e. including colostrum from all udder quarters) were taken at first milking after birth into 15 mL (TINE Norwegian Dairies) and 50 mL test tubes (Falcon ™ Conical Centrifuge tubes, Corning, NY, USA), respectively. Farmers were asked to report whether the cow had been suckled prior to sampling. The colostrum samples were frozen (− 18 °C) as soon as possible after sampling. For each sample, the farmer noted cow-specific information in a form provided by the researchers (Table 2).

**Table 2:** Cow-level variables and associated IgG levels in 167 colostrum samples from 19 dairy herds in a specific region in Norway (Ringsaker municipality, Hedmark County)

Variable	Class or mean (SD)	Herds (N)	Samples (n)	IgG, g/L (SD)
Parity	1	19	53	41.5 (23.44)
	2	17	46	34.1 (18.98)
	3	18	31	40.8 (25.48)
	> 3	17	37	47.9 (26.51)
Udder emptied at (first) milking?	Yes	19	101	44.3 (24.30)
	No	18	62	39.1 (23.29)
	Missing	4	4	26.8 (21.54)
Length of dry period (days)*^a^	67.0 (20.93)	19	112	40.3 (24.04)
	Missing	19	55	41.6 (23.28)
Time from calving to sampling (milking; h)*^a^	3.5 (3.93)	16	120	42.1 (24.82)
	Missing	3	47	37.3 (20.56)
Milk yield at first milking (L)	5.0 (2.71)	19	163	41.5 (23.31)
	Missing	4	4	19.8 (12.52)
Calving occurred in	Individual calving pen	9	59	40.4 (18.80)
	Group calving pen	2	9	42.7 (25.72)
	Loose housing	10	38	37.6 (18.31)
	Tie stall	3	25	48.9 (33.10)
	Other	10	28	35.1 (22.64)
	Missing	1	8	51.25 (39.62)
Calving ease	Spontaneous	17	110	40.9 (24.65)
	Easy pull	12	41	42.2 (22.22)
	Hard	3	3	54.0 (32.51)
	Missing	5	13	31.3 (17.37)
Antibiotic treatment at dry off?*	Yes	6	9	46.1 (33.14)
	No	15	71	41.3 (25.02)
	Missing	19	87	39.7 (21.68)
Leakage of colostrum prior to calving?*^a^	Observed	17	42	35.2 (19.98)
	Not observed	17	104	42.9 (24.52)
	Missing	2	21	41.1 (25.95)

Variables with > 10% missing entries (*) were excluded from the mixed multivariable model examining possible risk factors for low colostrum quality

^a^Variables that were found to explain variation in IgG in univariable analyses at P < 0.2

### Quantification of Brix%

The second farm visit took place once the sampling period was over. Project personnel assembled the collected colostrum samples. Subsequently, and after thawing in a project-personnel refrigerator, the indirect quantification of IgG by use of a digital refractometer (Milwaukee Digital Brix refractometer MA 871, Milwaukee Instruments, Inc., Rocky Mount, NC, USA) was performed on one aliquot (i.e. the 50 mL test tubes) of the samples according to the manufacturer’s instructions.

### Quantification of IgG (g/L)

The other aliquot (i.e. the 15 mL test tubes) of the colostrum samples were submitted in cool, insulated boxes to the TINE Mastitis Laboratory in Molde, Norway for analyses: RID (Triple J Farms, Bellingham, WA, USA) was used to determine IgG concentration (g/L).

### Statistical analysis

Data on IgG, Brix%, herd and cow level information was entered into Excel. Further data handling and statistical analyses were performed in Stata (Stata SE/14, Stata Corp., College Station, TX, USA).

### Agreement of RID vs. digital refractometer and diagnostic test evaluation

Diagnostic test characteristics were defined as follows: The sensitivity (Se) estimates the ability of the digital refractometer to correctly classify high quality colostrum (> 50 g/L by RID) and specificity (Sp) estimates the ability to correctly classify colostrum that is not of high quality (< 50 g/L by RID). Se and Sp at different cut-offs of Brix% were calculated using RID as gold standard, with > 50 g/L IgG indicating colostrum of high quality. Prior to calculation of Se and Sp, the RID and Brix% results from the colostrum samples were compared in 2 × 2 tables at Brix% cut-offs from 18 to 24. The optimal cut-off for Brix%, maximising the product of Se and Sp was estimated using the Liu method [18]. Brix% and colostrum IgG were compared by Pearson’s correlation coefficient.

### Factors associated with colostrum quality

To evaluate factors associated with colostrum quality we tested potential predictors from the questionnaire; herd level variables (Table 1) and cow level variables (Table 2). The outcome variable colostrum IgG (g/L) based on the RID assay was slightly right-skewed. It was therefore log-transformed for analysis purposes. To account for the hierarchical structure of the data, herd was entered as a random term. Univariable mixed linear regression was used as an initial screening; variables associated with colostrum IgG at a P-value < 0.2 were considered for further analysis. Additionally, predictors with > 10% missing entries were excluded from further evaluation, but data is shown for description. Subsequently, a multivariate linear mixed regression model was used for the investigation of possible associations between colostrum IgG and the selected predictors. Plausible interactions, established a priori (time from calving-sampling × calving occurred in, time from calving-sampling × calving ease, length of pasture period × pasture access, length of pasture period × pasture period, herd milk yield × concentrate (kg/days) fed to cows/heifers at day of expected calving) were also tested. The likelihood ratio test was used to evaluate the significance of categorical variables. Variables with P < 0.05 were retained in the final model and the only variable retained in the final model was parity. Any confounding effects by other fixed effects were evaluated by monitoring the estimates for the exposure of interest (colostrum IgG) as the potential confounders were included or removed from the multivariable model. Model assumptions were evaluated by visual inspection of normality plots of the residuals, and homoscedasticity was evaluated by plotting the predicted values against the residuals.

## Results

### Descriptive statistics

**Fig. 1:**
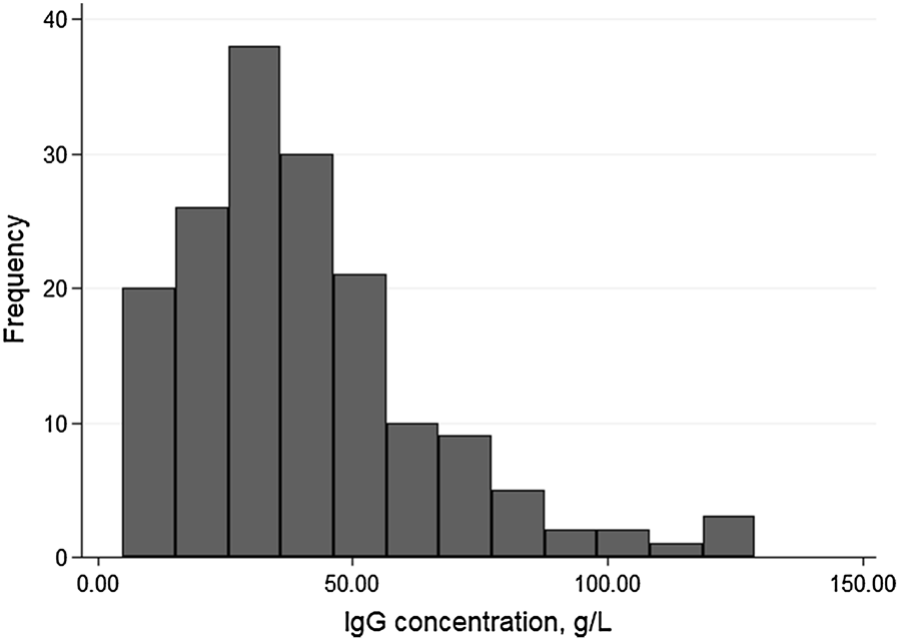
The distribution of IgG content (g/L) in colostrum samples from 167 Norwegian Red dairy cows in 19 herds

**Fig. 2:**
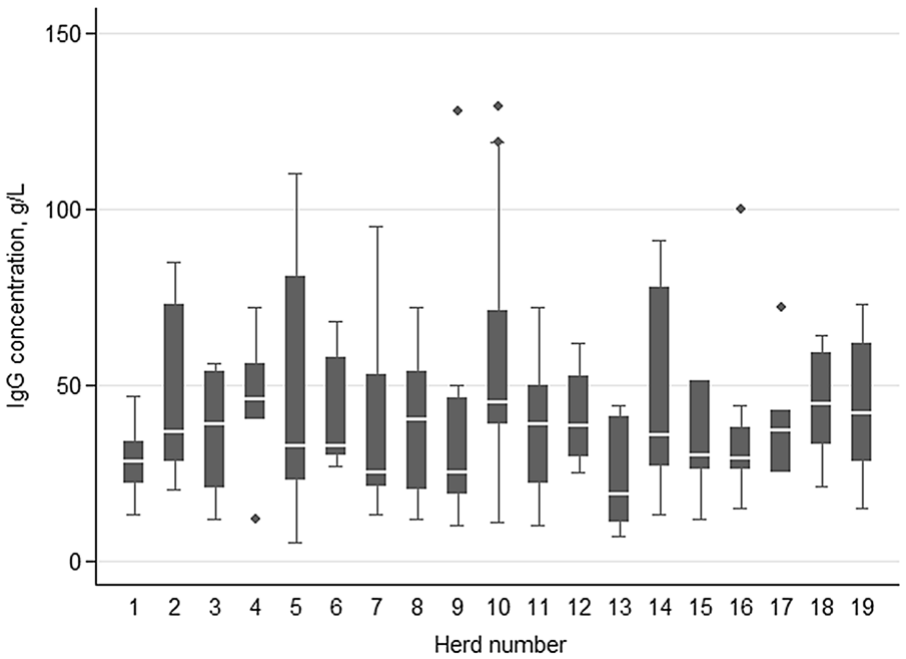
The variation in first-milking colostrum IgG among 19 herds (n = 167 dairy cows)

The final study sample consisted of 167 colostrum samples from 19 herds. Each herd contributed between 4 and 10 samples. The distribution of IgG content in the colostrum samples (prior to transformation of the variable for analysis) is shown in Fig. 1. Median colostrum IgG concentration varied among herds (Fig. 2). Overall, median colostrum IgG was 35.0 g/L and ranging from 5 to 129 g/L. Mean Brix% (± SD) was 19.7 ± 4.12% and ranging from 10.1 to 30.5. Results of descriptive analyses of herd- and cow-level predictors are shown in Tables 1 and 2, respectively. The majority of the colostrum samples (72.5%) had inferior quality as compared to the international standard of 50 g/L. Inferior quality colostrum samples were submitted from all herds.

### Diagnostic test evaluation

**Fig. 3:**
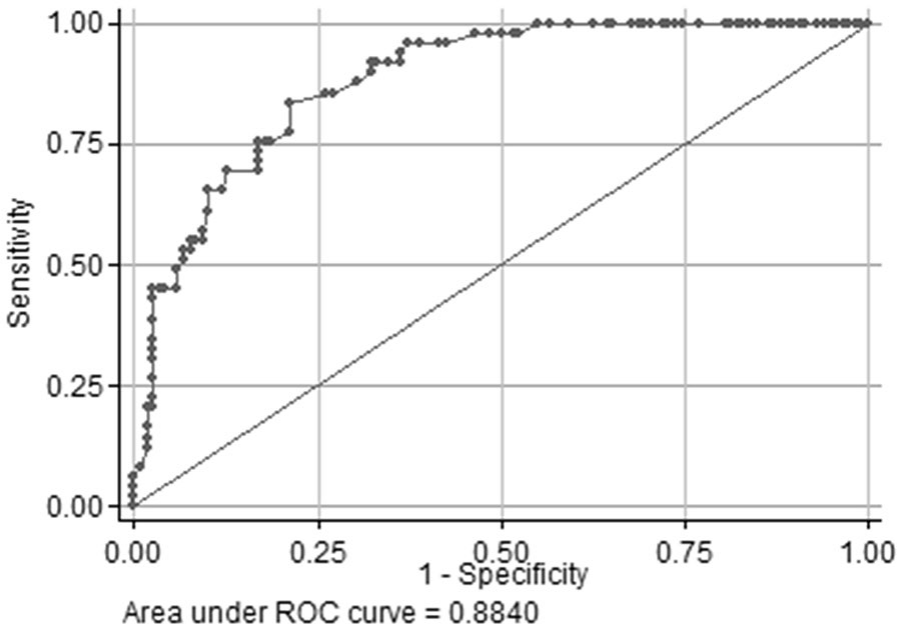
A receiver operating characteristic curve shows the relationship between clinical sensitivity and specificity for every possible cut-off in Brix% to identify the target condition of high quality colostrum (> 50 g/L) using radial immunodiffusion assay assessment as the gold standard in colostrum samples from 167 Norwegian Red dairy cows in 19 herds

**Fig. 4:**
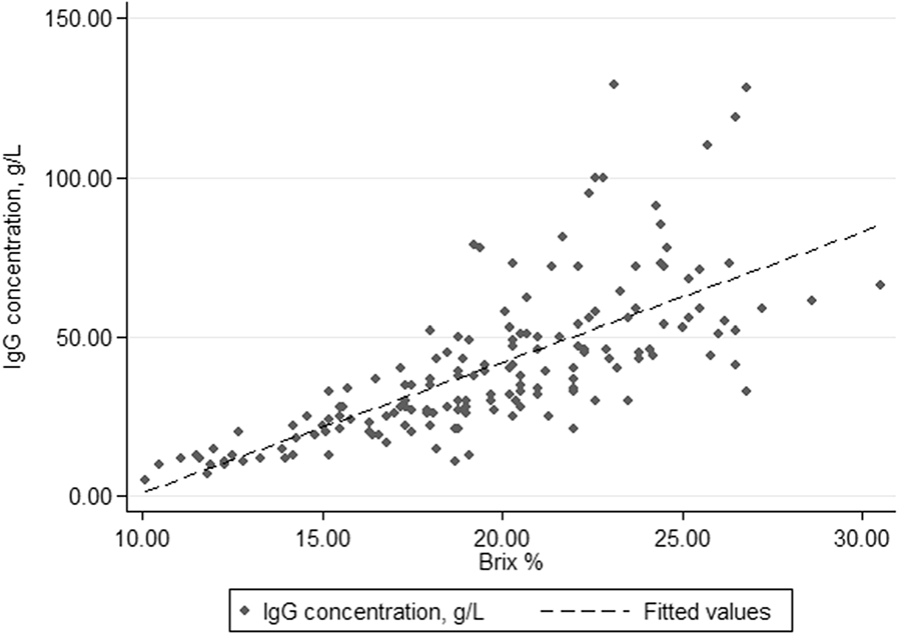
The association between IgG in bovine colostrum quantified directly, with the gold standard (single radial immunodiffusion) and indirectly using a handheld digital refractometer in colostrum samples from 167 Norwegian Red dairy cows in 19 herds

Observed counts of paired test outcomes per cut-off using RID and Brix are shown in Table 3. Se and Sp at different cut-offs of the Brix refractometer are shown in Table 4. The Sp increased and Se decreased with increasing Brix cut-off (Fig. 3). The cut-off of Brix maximizing the product of Se and Se was 20.6%, resulting in a Se and Sp of 84% and 79%, respectively. Brix and IgG were positively correlated (r = 0.71, P < 0.001, Fig. 4).

**Table 3:** Observed counts of paired test (n = 167 colostrum samples) outcomes per Brix cut-off using single radial immunodiffusion (RID) and Brix% refractometer (BRIX)

Brix cut-off	Test outcome RID/BRIX			
	High/high	High/low	Low/high	Low/low
18	49 (29%)	0 (0%)	65 (39%)	53 (32%)
19	47 (28%)	2 (1%)	49 (29%)	69 (41%)
20	45 (27%)	4 (2%)	38 (23%)	80 (48%)
21	38 (23%)	11 (7%)	25 (15%)	93 (56%)
22	34 (20%)	15 (9%)	20 (12%)	98 (59%)
23	27 (16%)	22 (13%)	10 (6%)	108 (65%)
24	22 (13%)	27 (16%)	5 (3%)	113 (68%)

High quality colostrum was defined as colostrum > 50 g/L and the results are shown as RID/BRIX, +/+, ± , ∓, −/−)

**Table 4:** Estimated sensitivity (Se) and specificity (Sp) for the digital refractometer at different cut-offs in Brix, 95% confidence intervals are given in brackets in 167 colostrum samples from 19 dairy herds in a specific region in Norway (Ringsaker municipality, Hedmark County)

	Brix 18%	Brix 19%	Brix 20%	Brix 21%	Brix 22%	Brix 23%	Brix 24%
Se	100.0 (92.7–100)	95.9 (86.0–99.5)	91.8 (80.4–97.7)	77.6 (63.4–88.2)	69.4 (54.6–81.7)	55.1 (40.2–69.3)	44.9 (30.7–59.8)
Sp	44.9 (35.7–54.3)	58.5 (49.0–67.5)	67.8 (58.6–76.1)	78.8 (70.3–85.8)	83.1 (75.0–89.3)	91.5 (85.0–95.9)	95.8 (90.4–98.6)

The target condition is identification of high quality colostrum (> 50 g/L) using radial immunodiffusion assay assessment as the gold standard

### Factors associated with colostrum quality

Descriptive statistics and univariate associations between herd- and cow-level explanatory variables and colostrum IgG are shown in Tables 1 and 2, respectively. Of the tested explanatory variables, parity was the only variable retained in the final model (Table 5). Colostrum from cows with 4 or more parities had the highest concentrations of IgG. Compared to colostrum from these, that of 2nd parity cows had a mean of 11.5 g less IgG/L: 29.4 (22.84–37.92) vs 40.9 (33.81–49.31), respectively. None of the other management factors at herd level were found to be associated with colostrum IgG. In the final model, the variation caused by the herd was negligible (data not shown). No major shortcomings were detected with respect to the evaluation of the model.

**Table 5:** Result estimates of the final mixed model describing risk factors for low colostrum quality in 167 colostrum samples from 19 dairy herds in a specific region in Norway (Ringsaker municipality, Hedmark County)

Variable	Class	Estimate	LSM	95% CI LSM		P
Intercept		3.71	40.9	–	–	< 0.001
Parity	1	− 0.14	35.5	27.74	45.47	0.252
	2	− 0.33	29.4	22.76	37.89	0.011
	3	− 0.19	33.8	25.67	44.45	0.191
	> 4	0	40.85	33.84	49.311	–

Lower and upper 95% confidence intervals (CI) are given for the back transformed least square means (LSM)

## Discussion

### Indirect quantification of IgG and diagnostic test evaluation

The correlation between the indirect measure of IgG (Brix) and IgG (quantified with RID) yielded results comparable to previous research [e.g. 8, 19]. This indicates that the digital refractometer provides a continuous, indirect measure of colostrum quality which corresponds well to IgG quantified by use of RID.

We found that the optimal empirical Brix cut-off to estimate colostrum samples with > 50 g/L IgG was 20.6%, which is comparable to that of another study: 20.9% [20]. Silva Del-Rio et al. [20] found that Se and Sp at this cut-off was 87.8% and 100%, respectively. In our study, the optimal empirical cut-off returned low Se and Sp of 84% and 79%, respectively, and thus does not provide the farmer with accurate information needed to take an informed decision. A recent meta-analysis identified Brix 22% as the optimal cut-off to identify high quality colostrum [11]. In our material, the Se, but not the Sp, was lower at this cut-off (69.4% vs. 82.0%) as compared to the eight meta-analyzed studies. Rather than maximization of Se and Sp, which might not be optimal due to the relative importance to correctly identify colostrum of low quality, a cut-off in Brix should preferably show a high Sp. The results of our study indicate that colostrum with > 23 Brix% (with > 90% Sp) indicates colostrum of high quality beyond reasonable doubt. Although the Se at this cut-off is low (55%), which potentially may lead to wastage of high quality colostrum, the relative importance may be less since colostrum usually is produced at sufficient volumes.

The results of the study indicate a need for further research to validate the refractometer for Norwegian conditions. The on-farm interpretation of the digital refractometer is linked to which cut-offs in Brix that indicate whether colostrum is of good quality with reference to the international standard of > 50 g/L IgG. However, the direct adaptation of this standard may not be optimal. Firstly, as pointed out by Buczinsky et al. [11], dichotomizing the quality of colostrum quality may be debatable. Secondly, the 50 g/L cut-off may not be relevant to identify colostrum of low quality under Norwegian conditions. The IgG content as quantified with RID was well below that of other international studies [15, 19, 21, 22]. Consequently, the prevalence of high quality colostrum (> 50 g/L IgG) in our study was much lower than that of other studies: 27.5 vs. 67.3–92.3 [11]. On the other hand, the colostrum quality in our study was comparable to recent Norwegian studies [13, 14]. Brix levels were also marginally lower (19.7 vs. 20. 7%) than what was shown by [23], but much lower than 24–28% reported by others [8, 24].

This is the third study documenting that the majority of the colostrum samples from Norwegian Red dairy cows are of inferior quality in terms of IgG concentration when compared to international guidelines. Previous studies have demonstrated increased colostral IgG levels in response to routine vaccination [25]. We speculate that the lacking tradition to routinely vaccinate dairy cows in Norway might contribute to these low IgG levels. In addition, Norway is declared free of many of the major infectious diseases, including infectious bovine rhinotracheitis and bovine viral diarrhea [26]. Despite the relatively low colostrum IgG levels, calf mortality is not high relative to other countries [27] which supports the idea that a low colostrum IgG level may reflect the natural situation rather than a pathological one. Future studies should focus on determining a cut-off for colostrum IgG level for Norwegian cows at which calves attain sufficient serum IgG to prevent disease. The farmers were instructed to report whether the cow had been suckled prior to sampling, and this was not the case for any of the samples. However, first milking and sampling occurred at a mean of 3.5 h after parturition. Consequently, although measures were taken to prevent it, we cannot exclude that some of the cows suckled prior to sampling.

### Factors affecting colostrum quality

The quality of colostrum is dependent on other factors than just IgG. A high hygienic quality is pivotal for a successful intestinal absorption [28]. Live maternal cells, growth factors, cytokines and nutrient composition per se are also important determinants of colostrum quality [28]. In our study, colostrum IgG was found to vary both within and between herds although the residual variation explained by herd in the final model was negligible. Between-herd variation has been described in earlier papers, both in Norway [13, 14], and internationally [23]. Differences between herds linked to environmental, management and nutritional factors known to affect colostrum quality likely account for this variation [13].

Parity was the only factor that influenced colostrum IgG concentration in this study. Compared to colostrum from cows with 4 or more lactations, colostrum from cows in the second lactation was found to have the lowest IgG values. Similar findings have previously been described by Gulliksen et al. [13] and Johnsen et al. [14]. The results may point to a shortcoming in the management of these cows which should be addressed in future research. Other Norwegian studies have found lower colostral IgG levels during the winter [13, 14]. Consequently, it is of prime importance for producers to check colostrum quality during the winter in general, and for second parity cows specifically.

Dry period length, time from calving to sampling and observation of colostrum leakage prior to calving are factors which are known to influence colostrum quality. Although these measures were found to influence colostrum IgG in our study, the factors were not included in the final model due to many missing entries. Leakage of colostrum may be associated with a risk of a low colostrum quality [29]. It was therefore advised that farmers observing colostrum leakage during parturition should harvest colostrum as soon as possible. A long time-lag between birth and colostrum harvest by the farmer is another known risk factor of low colostrum quality which is in accordance with our results. In our study, this time-lag ranged from 0 to 20 h (data not shown) indicating a potential to minimize this effect by closer surveillance of calving. Omitting the dry period, or drastically shortening it (< 30 days) is shown to result in lower colostrum IgG [30]. Our data suggested the same reciprocal relationship between these two variables, although missing entries prevented us from concluding on this matter.

No factors related to feeding management were found to influence colostrum IgG. A low sample size and the fact that these factors were registered at herd level, may explain the lack of associations. Gulliksen et al. [13] identified a negative correlation between concentrate intake and colostrum IgG which suggested associated risks with a high energy diet. However, variation in e.g. daily increment of concentrate allowance in the weeks prior to calving was low among the herds in the current study. Most farmers fed concentrate at around 2 weeks prior to calving, and most herds feed 1–3 kg of concentrate at the time of expected calving to both heifers and dry cows. Limitations of the study are related to the small study sample which was drawn from a specific region in Norway. These aspects may have artificially deflated the variation in management between the herds.

## Conclusion

The agreement between colostrum IgG and Brix% is good. However, the diagnostic test evaluation indicates suboptimal performance in identifying high vs. low colostrum quality in this population. This may be related to a much higher proportion of the samples with < 50 g/L IgG. The only factor found to be associated with low colostrum quality was parity. Specifically, cows in the second parity were found to produce colostrum with lower quality.

## Data Availability

The datasets used and analysed during the current study are available from the corresponding author on reasonable request.

## Abbreviations

FPT: failure of passive transfer
IgG: immunoglobulin G
RID: single radial immunodiffusion
Sp: specificity
Se: sensitivity
